# Congenital spongiform leukodystrophy in 2 female littermate German shepherd puppies

**DOI:** 10.1111/jvim.17055

**Published:** 2024-03-27

**Authors:** Ricardo De Miguel, Devon Wallis Hague, Jennifer L. Johnson, Amber M. Zilinger, Anna Kukekova, Stephane Lezmi

**Affiliations:** ^1^ AnaPath Services GmbH Liestal Switzerland; ^2^ Department of Veterinary Clinical Medicine University of Illinois Urbana Illinois USA; ^3^ Department of Animal Sciences, College of Agricultural, Consumer and Environmental Sciences University of Illinois at Urbana‐Champaign Champaign Illinois USA; ^4^ Department of Pathobiology University of Illinois at Urbana‐Champaign Champaign Illinois USA; ^5^ Excilone Services Jouy‐en‐Josas France

**Keywords:** ASPA gene, aspartoacylase, Canavan disease, myelin

## Abstract

Two 9‐week‐old female littermate German Shepherd puppies showed severe high‐frequency low‐amplitude trembling that worsened with movement. The white matter (WM) of the central nervous system (CNS) showed bilateral diffuse severe spongiosis in the cerebellum, brainstem, spinal cord, and the neuropil of the oculomotor and red nuclei. The cortical corona radiata was less severely affected. Rare necrotic or apoptotic glia‐like cells also were identified in the WM. Luxol fast blue staining disclosed severe diffuse myelin loss in the entire CNS; peripheral nerves were spared. Glial fibrillary acidic protein immunohistochemistry showed diffuse astrogliosis and astrocytosis in the WM. Genetic analyses of the littermates excluded the aspartoacylase (ASPA) gene as a candidate for this condition in dogs. In conclusion, this description of a rare congenital spongiform leukodystrophy in the German Shepherd breed, closely resembling to Canavan disease in humans, is likely caused by a genetic alteration unrelated to the ASPA gene.

**Abbreviations**
ASPA gene: aspartoacylase gene
CNS: central nervous system
GFAP: glial fibrillary acidic protein
SNP: single nucleotide polymorphism
UCSC: University of California, Santa Cruz
WM: white matter

## 1 INTRODUCTION

Leukodystrophies are rare insidious genetic diseases caused by inherent defects in the synthesis and maintenance of myelin, which generally leads to its progressive degeneration after birth.^1^, ^2^ The neuropathology of these disorders is characterized by bilateral symmetric involvement of white matter (WM) with a distinct disease‐specific distribution pattern of lesions.^3^ Depending on the lesion distribution, leukodystrophies can lead to a plethora of clinical signs related to movement, vision, hearing, balance, ability to eat, and behavior.^1^, ^4^

In animals, many myelin disorders have been reported and they mainly are characterized by early onset in life and progressive worsening.^1^, ^2^ In dogs, genetic basis for dysmyelinating disorders usually is suspected and causative genes for myelination defects have been identified in several breeds.^5^, ^6^, ^7^, ^8^, ^9^, ^10^ In human medicine, spongy leukodystrophy (also known as Canavan disease) is characterized by diffuse symmetrical WM spongy degeneration with prominent early involvement of subcortical U fibers and is caused by mutations in the aspartoacylase (ASPA) gene.^11^, ^12^ Our aim was to describe a Canavan‐like disease in 2 female littermate German Shepherd puppies and investigate the ASPA gene as a candidate for this disorder.

## 2 MATERIALS AND METHODS

### 2.1 History and clinical examination

Two 9‐week‐old female littermate German Shepherd puppies were referred to the Veterinary Teaching Hospital of the University of Illinois with a history of progressive tremors present since birth. Both animals belonged to a 6‐member litter composed of 2 affected females and 4 unaffected males. The dam was primiparous whereas the sire had a previous 3‐animal litter composed of 1 stillborn and 2 puppies of unknown sex that died 3 hours and 3 days after birth, respectively.

Clinical examinations including body weight and neurologic evaluation were performed. Animals were euthanized because of clinical worsening and poor prognosis.

### 2.2 Pathology

After a systematic necropsy evaluation, tissues were sampled and fixed in 10% neutral‐buffered formalin. Central nervous system (CNS) tissue was trimmed at 5‐mm intervals. Samples were routinely processed for paraffin embedding and 4‐μm sections were stained with hematoxylin‐eosin (HE). Additionally, Luxol fast blue staining and immunohistochemistry (IHC) for glial fibrillary acidic protein (GFAP) were performed in all sections of the CNS. An age‐matched normal n dog brain was used as control.

For IHC, samples were subjected to pretreatments for antigen retrieval (30 minutes in a solution of 10 mM citric acid pH 6 immersed in a water bath at 95°C), endogenous peroxidase inhibition, and nonspecific binding sites blockade (20 minutes in normal horse serum diluted 1:200 in phosphate buffer saline [PBS]). Tissue samples then were incubated overnight at 4°C with a specific polyclonal antibody against glial fibrillary acidic protein (Anti‐GFAP, Ref: Z0334, DAKO), labeled for 30 minutes at room temperature with anti‐mouse EnVision HRP System (DAKO, Agilent Technologies) and revealed with 3,3′‐diaminobenzidine. The specificity of the technique was controlled by immunolabeling of brain tissue of a normal brain, which was used as control.

### 2.3 Genetic analyses

The DNA was extracted from 2 affected German Shepherd littermates (AS‐1 and BS‐7), their normal parents, 6 normal German Shepherd dogs, and 1 dog of unknown breed (13‐CC; Table S2). All DNA samples were extracted from blood except AS‐1, BS‐7, and 13‐CC, for which DNA samples were extracted from tissues fixed in paraffin. For DNA extraction from paraffin, an approximately 3‐mm block of tissue was dissected from the paraffin. Paraffin was removed by putting the tissue in 1 mL of xylene, vortexing it for 10 seconds, and then microcentrifuging at 14 000 rpm for 2 minutes and removing the xylene, containing dissolved paraffin, as waste. This procedure was repeated 5 times. The samples then were submerged in 1 mL 100% ethanol, vortexed for 10 seconds, and centrifuged at 14 000 rpm for 2 minutes, removing the ethanol that contained the remaining xylene. The procedure was repeated 3 times. The tissue then was air‐dried for 10 minutes, and DNA was extracted using the QIAamp DNA Mini kit (Qiagen, Hilden, Germany) following the protocol for DNA tissue extraction.

The PCR primers for each of 6 exons of the *ASPA* gene were designed (Table S1) using the CanFam3.1 genome assembly (the University of California, Santa Cruz Genome Browser). The *ASPA* exons were amplified by conventional PCR using GoTaq DNA Polymerase (Promega, Madison, WI). The samples were amplified in 50 μL PCR reactions under the following conditions: 96°C for 2 minutes; 30 cycles of 96°C (20 seconds), 58°C (20 seconds), and 72°C (20 seconds); and a final extension at 72°C for 5 minutes. The PCR products were purified using QIAquick PCR Purification kit (Qiagen) and sequenced on an ABI3730 DNA Analyzer (PE Biosystems, Foster City, CA). Sequencing results were analyzed using Sequencher version 5.1 sequence analysis software (Gene Codes Corporation, Ann Arbor, Michigan; http://www.genecodes.com). Sequenced PCR products obtained from 2 affected and 9 normal dogs were aligned against exons of the *ASPA* gene (CanFam3.1) and searched for polymorphisms.

## 3 RESULTS

### 3.1 Clinical examination

At clinical examination, marked growth retardation was observed in both animals. Affected females weighed 2.7 and 3.4 kg, which sharply contrasted with the weight of male littermates (range, 5.4‐5.6 kg). Neurologic examination of puppies showed high‐frequency and low‐amplitude tremors that worsened with movement (Video S1). When standing, animals showed wide‐based stance and rapid collapse. All withdrawal reflexes were present except for cutaneous trunci reflex, which could not be elicited. Normal placing responses were present in all 4 limbs. Neither hyperesthesia nor mentation abnormalities were detected. Presumptive diagnosis of a myelinopathy was established with hypomyelination or dysmyelination as possible differential diagnoses.

### 3.2 Pathology

**FIGURE 1:**
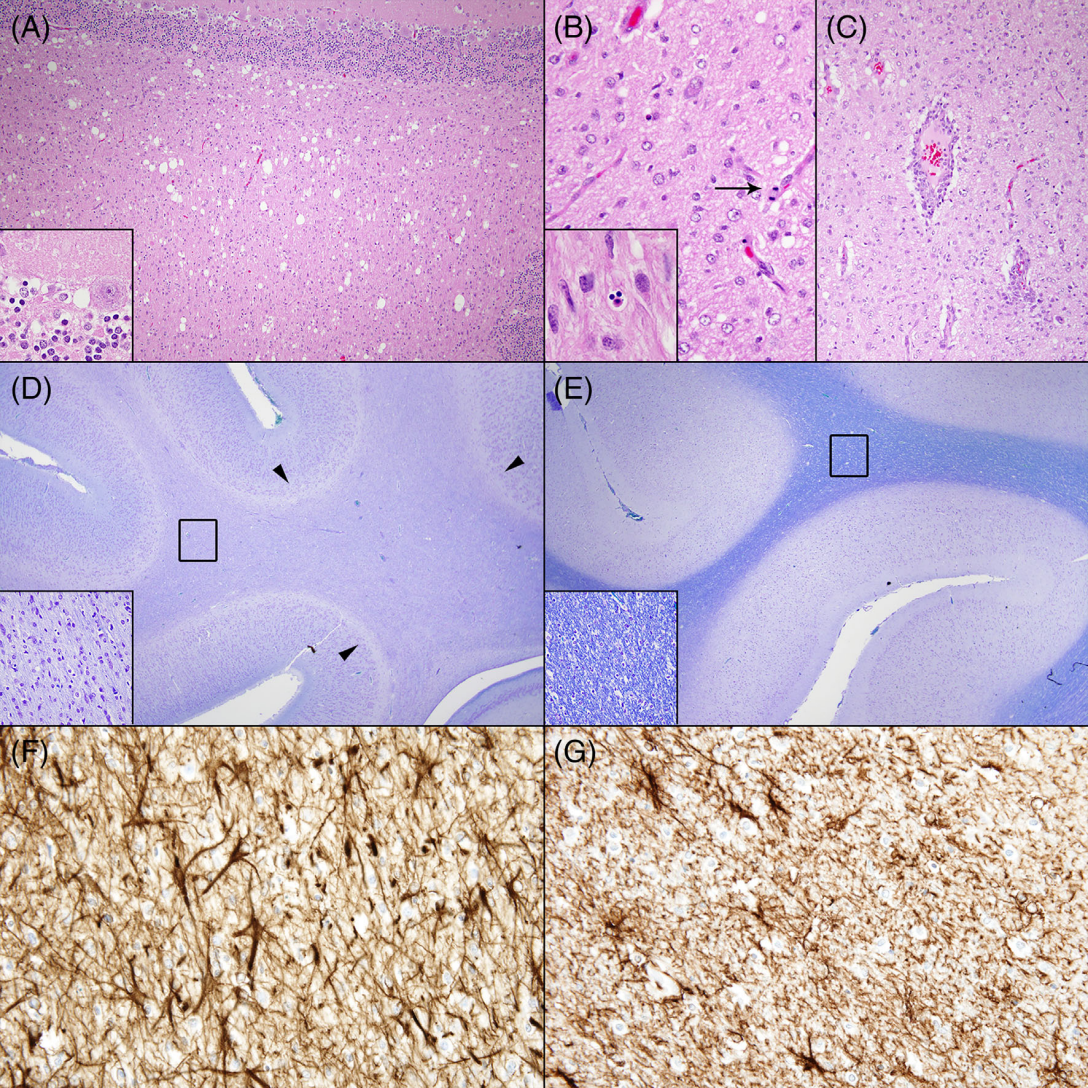
Spongiform leukodystrophy, CNS, dog. (A) Severe spongy vacuolation of the WM, cerebellum. Inset: Vacuolation of the neuropil of the cerebellar folia, boundary between granular and molecular layers. Hematoxylin and Eosin. (B) Moderate gliosis with rare mitotic figures (arrowhead). Inset: Occasional apoptotic or necrotic glial cells are seen. Hematoxylin and Eosin. (C) Mild to moderate lymphoplasmacytic perivascular cuffing in perilesional areas. Hematoxylin and Eosin. (D) Severe myelin loss in the WM of cerebral hemispheres with marked involvement of the subcortical U‐fibers (arrowheads). Inset: Higher magnification of the boxed (black) region. Affected German Shepherd. Luxol fast blue staining. (E) Normal myelination of the WM. Inset: Higher magnification of the boxed (black) region. Nonaffected age‐matched control dog. Luxol fast blue staining. (F) Severe astrogliosis and astrocytosis of the WM. Affected German Shepherd. Antiglial fibrillary acidic protein immunohistochemistry. (G) Normal astrocytes within the WM. Nonaffected age‐matched control dog. Antiglial fibrillary acidic protein immunohistochemistry.

**FIGURE 2:**
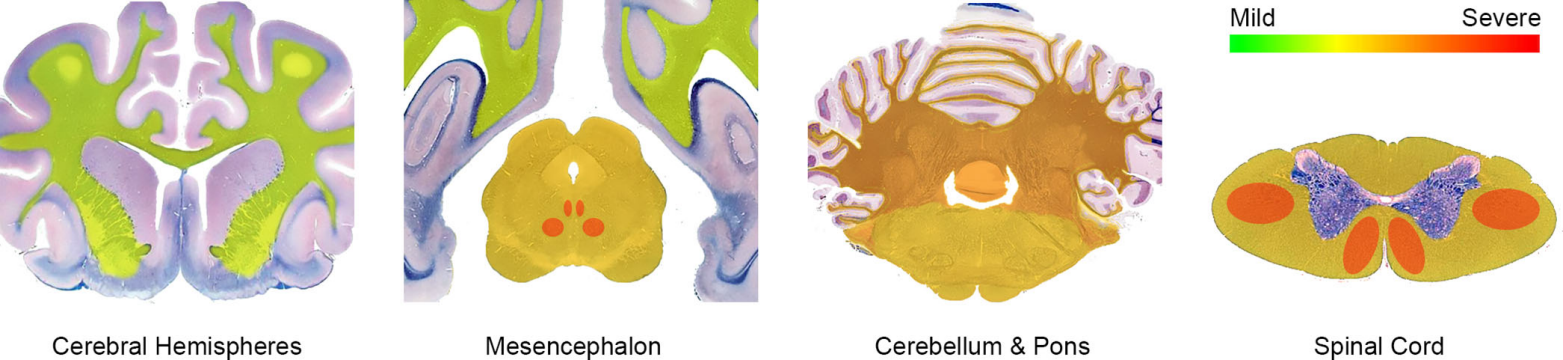
Distribution and severity of spongiform changes only. Color of the affected area signifies the severity and varies between green (mild severity) and, red (severe). Main affected areas were the WM of cerebellum, brainstem and spinal cord. Notably, the oculomotor and red nuclei in the mesencephalon and ventral and lateral funiculi in the spinal cord were severely affected. (*Histologic images were taken and modified from the 6120 Canine Brain Transections Website—College of Veterinary Medicine, University of Minnesota*).^15^

Gross examination of organs in both puppies was unremarkable. Microscopic examination identified the presence of bilateral diffuse vacuolation of the WM (ie, spongiosis) in both animals mainly affecting the cerebellum, brainstem, and spinal cord (especially the ventral and lateral funiculi; Figures 1 and 2). Brainstem nuclei (mainly the oculomotor and red nuclei; Figure 2) also showed marked vacuolation of the neuropil, without overt damage of the neuronal soma. Similar but less severe vacuolation was noted at the junction of the molecular and granular cerebellar cell layers, in the WM of the corona radiata in the cortex, and the peripheral segments of the optic nerves (Figure 2). Luxol fast blue staining identified severe and diffuse loss of myelin in the WM which was especially evident at the level of the subcortical U‐fibers and corona radiata of the cortex, and in all abovementioned neuroanatomical regions with higher severity of spongiosis (Figure 1D,E [control]).

These changes were associated with occasional single cell apoptosis or necrosis and rare mitotic figures within suspected glial cells in the WM (Figure 1B). Virchow‐Robin spaces of intralesional and perilesional blood vessels were expanded by few inflammatory cells, mainly lymphocytes and plasma cells (Figure. 1C). The WM also featured notable astrogliosis and astrocytosis as evidenced by the increased number of GFAP‐positive astrocytes and prominent astrocytic processes in the entire brain and spinal cord (Figure 1F,G [control]). Peripheral nerves showed no abnormalities or myelin loss. The final diagnosis was congenital spongy leukodystrophy (or leukoencephalomyelopathy).

### 3.3 Genetic analyses

Based on the history, clinical signs, and histopathology results, a genetic cause of the disease was suspected and the *ASPA* gene was tested as a candidate. Comparison of *ASPA* exonic sequences from 2 affected individuals, their nonaffected parents, and 7 normal dogs against canine reference sequence (CanFam3.1) identified 9 single nucleotide polymorphisms (SNPs). The SNPs 1, 3, and 8 were located in coding regions (coding exons 2, 3, and 6, respectively) whereas the remaining SNPs were located in introns (Table 1 and S2). Single nucleotide polymorphisms 1 and 3 were nonsynonymous, SNP 8 was a synonymous mutation. The analysis of amino acid changes caused by SNPs 1 (D104N) and 3 (N145T) with PolyPhen‐2 v2.2.2r398^13^ did not find any effect of these mutations on the ASPA protein (score of 0.000; sensitivity, 1.00; specificity, 0.00). The analysis of ASPA protein sequence on the UCSC Genome Browser found these amino acids to be evolutionarily polymorphic. Both affected littermates were heterozygous for all identified SNPs except 1 exonic SNP (SNP 8, synonymous polymorphism in exon 6) and 2 noncoding SNPs (SNP 2 located in intron 2 and SNP 9 located in 3′ untranslated region; Table 1). Haplotype analysis of identified SNPs established 6 *ASPA* haplotypes in sequenced dogs (Table S3). Both affected littermates were heterozygous for haplotypes *b* and *c*, whereas their sire was heterozygous for *a* and *b* and the dam was heterozygous for *a* and *c* (Table 1). Under the assumption that congenital spongy leukodystrophy described in our study has a recessive mode of inheritance, the genotyping results allow us to exclude the *ASPA* gene as a candidate for this disease.

**TABLE 1:** ASPA genotypes and haplotypes identified in sequenced dogs.

	SNP ID	Region	Genotype	Sire	Dam	Affected puppies (N = 2)	Non‐aff. unrelated GSD (N = 6)	Non‐aff. other breed (N = 1)
Genotype	SNP1	Exon 2	AA	—	—	—	—	—
			GA	—	x	100%	—	x
			GG	x	—	—	100%	—
	SNP2	Intron 2	AA	—	—	100%	16.7%	x
			GA	x	x	—	33.3%	—
			GG	—	—	—	50%	—
	SNP3	Exon 3	AA	—	—	—	16.7%	x
			AC	x	—	100%	33.3%	—
			CC	—	x	—	50%	—
	SNP4	Intron 3	AA	—	—	—	—	—
			GA	—	x	100%	—	—
			GG	x	—	—	100%	x
	SNP5	Intron 3	CC	—	x	—	50%	—
			CT	x	—	100%	33.3%	x
			TT	—	—	—	16.7%	—
	SNP6	Intron 4	CC	—	—	—	16.7%	—
			CT	x	—	100%	33.3%	x
			TT	—	x	—	50%	—
	SNP7	Intron 4	AA	—	—	—	16.7%	—
			GA	x	—	100%	33.3%	x
			GG	—	x	—	50%	—
	SNP8	Exon 6	CC	—	—	—	—	—
			CT	—	—	—	16.7%	—
			TT	x	x	100%	83.3%	x
	SNP9	3′ UTR	CC	—	—	100%	16.7%	x
			CT	x	x	—	66.67%	—
			TT	—	—	—	16.7%	—
Haplotype			*aa*	—	—	—	16.7%	—
			*bb*	—	—	—	16.7%	—
			*ab*	x	—	—	33.3%	—
			*ac*	—	x	—	—	—
			*bc*	—	—	100%	—	—
			*ad*	—	—	—	16.7%	—
			de	—	—	—	16.7%	—
			*bf*	—	—	—	—	x

*Note*: Analyzed animals included the sire, the dam, their affected puppies, 6 nonaffected and unrelated German shepherd dogs and 1 unrelated dog from another breed. Frequency of each genotype/haplotype is expressed as percentage (%). The nonsynonymous SNPs are underlined. The genotypes and haplotypes identified for parents and the nonaffected dog of an unknown breed are marked as “x.”

## 4 DISCUSSION

Primary myelinopathies encompass several conditions that develops as a consequence of affected myelin sheets and are broadly divided into 3 overlapping categories: disorders associated with absent or retarded myelination (hypomyelinogeneses), conditions related to inherent defects on myelin structure that leads to myelin loss (leukodystrophies), and entities characterized by dramatic vacuolation of myelin without myelin breakdown (spongy myelinopathies).^1^ In our cases, the marked spongy vacuolation of WM was associated with severe loss of myelin, thus a primary spongy myelinopathy was eliminated. Furthermore, the early onset of the disease together with the progressive worsening of clinical signs associated with reactive gliosis, occasional cellular necrosis or apoptosis of glia‐like cells, and mild lymphohistiocytic perivascular cuffing suggested degeneration of myelin rather than primary hypomyelinogenesis.

In humans, Canavan disease mainly affects the WM of cerebral hemispheres, cerebellum, corpus callosum, optic tracts, brainsteam, and spinal cord.^12^, ^14^ This distribution highly aligned with the affected areas found in our cases. Additionally, Canavan disease is characterized by blurring of the cortical gray‐WM junction because of U‐fiber demyelination and a prominent spongy change in the boundary between the molecular and granular cerebellar cell layers in the cerebellum.^12^ Both aforementioned changes also were observed in these German Shepherd puppies. Unlike Canavan disease, involvement of the oculomotor and red nuclei in the midbrain explains the tremors observed in the puppies, which were not as prominent as those observed in humans with spongy leukodystrophy.^15^ In this sense, lesions in the lateral and ventral funiculus also could contribute to dysfunction of motor signals and points toward potential descending damage through the rubrospinal tract. Canavan‐like disease recently has been reported in cats associated with disruption of oligodendrocyte homeostasis and vacuolation in the CNS. However, gray matter was more affected than WM in these animals, which contrasts with the distribution of lesions in humans and the puppies of our study.^16^

Canavan disease is an autosomal recessive disorder caused by loss‐of‐function mutations in the ASPA gene.^11^ This enzyme is constitutively expressed in oligodendrocytes and plays a key role in the catabolism of *N*‐acetylaspartate and *N*‐acetylaspartylglutamate.^14^ Although the exact pathogenesis of spongy leukodystrophy in humans remains to be elucidated, *N*‐acetylaspartate accumulation has been related to an impairment of myelin synthesis and maintenance as well as to an induction of oligodendrocyte toxicity.^17^ Genetic screening of the *ASPA* gene in both affected and nonaffected German Shepherd dogs identified 9 polymorphisms. Single nucleotid polymorphims 1 and 3 were located in coding regions of the *ASPA* gene and lead to amino acid changes, however, it is unlikely that they can cause the disease because of their low impact on protein function, low evolutionary conservation, and the fact that affected dogs were heterozygous for both SNPs. If the spongy leukodystrophy in these German Shepherd dogs segregates as an autosomal recessive trait, the *ASPA* gene can be excluded.

Although considered rare, several myelin disorders have been reported, mainly in purebred dogs. Hypomyelinating leukodystrophies have been described in German shepherd dogs and are associated with similar clinical signs (tremors) but different pathophysiology (ie, hypomyelination rather than oligodendrocyte damage and degeneration of myelin) and histopathological features (ie, lack of perivascular cuffing, lack of single cell necrosis or apoptosis of glia‐like cells and less severe vacuolation of the WM) than in the puppies of our study.^9^ Moreover, a genetic predisposition was strongly suspected but genetic analysis was inconclusive.^9^ A similar condition was described in Standard Schnauzers related to a missense mutation in the *TSEN54* gene.^10^ Widespread vacuolation of the WM with minimal myelin loss has been associated with a missense mutation in the mitochondrial cytochrome b gene in Australian cattle dogs and Shetland sheepdogs.^18^ Cerebellar ataxia of Malinois puppies is a congenital disease, the genetic cause of which has been suspected.^19^ Recently, the characteristic spongy degeneration of cerebellar WM and gray matter has been linked to different mutations in the *ATP1B2* and *KCNJ10* genes.^7^, ^8^ Krabbe disease is a well‐known leukodystrophy caused by a defined mutation in the GALC gene in Cairn and West‐Highland white terriers.^1^ Involvement of these genes seems an unlikely cause of the spongy leukodystrophy in these German Shepherd puppies because of clinicopathological differences. Furthermore, other leukodystrophies and diseases characterized by spongy degeneration with a highly suspected but nonproved genetic trait have been reported in different purebred dogs such as Dalmatians, Afghan hounds, Rottweilers, Samoyeds, Labrador Retrievers, Silky Terriers, and Shetland sheepdogs.^1^, ^2^, ^20^, ^21^, ^22^, ^23^

Infectious processes such as parvoviral infection and toxic causes such as rodenticide bromethalin intoxication also can be related to spongy degeneration of the CNS in dogs.^24^, ^25^ These causes together with metabolic conditions (eg, hepatic encephalopathy, citrullinemia, and asparagine synthetase deficiency) were considered in our differential diagnosis but ruled out in the light of clinical history and histopathologic findings.

In conclusion, our report provides a description of a congenital spongiform leukodystrophy in the German Shepherd breed. This condition resembles its counterpart in humans, Canavan disease. The precise etiology of this Canavan‐like spongiform leukodystrophy in German Shepherd dogs remains unclear, but a genetic basis is highly suspected.

## CONFLICT OF INTEREST DECLARATION

Authors declare no conflict of interest.

## OFF‐LABEL ANTIMICROBIAL DECLARATION

Authors declare no off‐label use of antimicrobials.

## INSTITUTIONAL ANIMAL CARE AND USE COMMITTEE (IACUC) OR OTHER APPROVAL DECLARATION

Authors declare no IACUC or other approval was needed.

## HUMAN ETHICS APPROVAL DECLARATION

Authors declare human ethics approval was not needed for this study.

## Supporting information


**Data S1.** Supporting Information.

**Video S1.** Main clinical signs were characterized by severe tremors worsening with movements and wide‐based stance.
