# Supplementary material for: Congenital spongiform leukodystrophy in 2 female littermate German shepherd puppies
Source: J Vet Intern Med. 2024 Mar 27;38(3):1730–6. doi: 10.1111/jvim.17055 (PMC11099797; doi:10.1111/jvim.17055)
Supplement: Supplementary file 1 — Data S1. Supporting Information. [file JVIM-38-1730-s001.docx]

**Supplemental Table 1**. Primers used for PCR amplification of several regions of the aspartoacylase (*ASPA*) gene . Start and end primer positions are indicated according to CanFam3.1 assembly.

| **NAME** | **DIRECTION** | **SEQUENCE** | **LOCATION** | **START** | **END** | **Position in ASPA** |
| --- | --- | --- | --- | --- | --- | --- |
| aspa-f1 | Forward | GAACTCTGCACTTTGCACTTTGG | **Chr.** 9 | 47343139 | 47343161 | Intron before Exon1 |
| aspa-r1 | Reverse | ACCCATACATGTTCTCATTCACA | **Chr.** 9 | 47343568 | 47343590 | Intron after Exon1 |
| aspa-f2a | Forward | AAAGTTTTGGCAACTGGCTCCT | **Chr.** 9 | 47346829 | 47346850 | Intron before Exon2 |
| aspa-r2a | Reverse | GGACCCCTTCTTCTGACCCTTT | **Chr.** 9 | 47347205 | 47347226 | Intron after Exon2 |
| aspa-f3j | Forward | TGGCAAAGAGAACAAAGTGTATGG | **Chr.** 9 | 47348520 | 47348543 | Intron before Exon3 |
| aspa-r3j | Reverse | AAGCCATAATGATAAGCAAGGTAAC | **Chr.** 9 | 47348763 | 47348787 | Intron after Exon3 |
| ASPA-F3h | Forward | TCCTGTGATGCTACATGGTCTACCT | **Chr.** 9 | 47352285 | 47352309 | Intron before Exon 4 |
| ASPA-R3h | Reverse | TGAGGAATGGCTGAAGTGAATAACA | **Chr.** 9 | 47352687 | 47352711 | Intron after Exon 4 |
| Exon5-5'F | Forward | CATCTATCCTTTAGTGTGTTTGTGC | **Chr.** 9 | 47356328 | 47356352 | Intron before Exon 5 |
| Exon5-5'R | Reverse | CCTGCAGATTAGGGTGGATCAG | **Chr.** 9 | 47356638 | 47356659 | Exon 5, 3' side |
| Exon5-3'F | Forward | GGAAAAGAATTTCCTCCTTGTGC | **Chr.** 9 | 47356548 | 47356570 | Exon 5, 5' side |
| ASPA-R4b | Reverse | TTCTTTTTCAAAGTTGGGTCTCA | **Chr.** 9 | 47356697 | 47356719 | Intron after Exon 5 |
| ASPA-F5 | Forward | AGCAAGAGGGAAGACCCAAATG | **Chr.** 9 | 47360369 | 47360390 | Intron before Exon 6 |
| ASPA-R5 | Reverse | GTGCAAGGCACTGGACTAGGTG | **Chr.** 9 | 47360713 | 47360734 | Intron after Exon 6 |

**Supplemental Table 2.** Dog samples used for PCR amplification and sequencing and identified ASPA polymorphisms and genotypes. Single nucleotide polymorphism (SNP) locations on dog chromosome 9 are indicated according to CanFam3.1. Non-synonymous polymorphisms are marked in blue.

|  |  |  |  |  | **HAPLOTYPE** | **GENOTYPE** | | | | | | | | |
| --- | --- | --- | --- | --- | --- | --- | --- | --- | --- | --- | --- | --- | --- | --- |
|  |  |  |  |  |  | **SNP1** | **SNP2** | **SNP3** | **SNP4** | **SNP5** | **SNP6** | **SNP7** | **SNP8** | **SNP9** |
|  |  |  |  |  |  | exon 2 | intron 2 | exon 3 | intron 3 | intron 3 | intron 4 | intron 4 | exon 6 | 3` UTR |
| **Dog ID** | **Breed** | **Disease Status** | **Tissue** | **Sample type** |  | 47,347,003 | 47,348,553 | 47,348,584 | 47,348,716 | 47,348,746 | 47,356,406 | 47,356,407 | 47,360,601 | 47,360,624 |
| Sire | GSD | Unaffected | Blood | Frozen | *ab* | GG | GA | AC | GG | TC | CT | GA | TT | CT |
| Dam | GSD | Unaffected | Blood | Frozen | *ac* | GA | GA | CC | GA | CC | TT | GG | TT | CT |
| Pup AS-1 | GSD | Affected | Spleen | Paraffin | *bc* | GA | AA | AC | GA | TC | CT | GA | TT | CC |
| Pup BS-7 | GSD | Affected | Spleen | Paraffin | *bc* | GA | AA | AC | GA | TC | CT | GA | TT | CC |
| 13-CC | Unkn. | Unaffected | Brain | Paraffin | *bf* | GA | AA | AA | GG | TC | CT | GA | TT | CC |
| Rin | GSD | Unaffected | Blood | Frozen | *ad* | GG | GG | CC | GG | CC | TT | GG | TT | CT |
| Reya | GSD | Unaffected | Blood | Frozen | *de* | GG | GG | CC | GG | CC | TT | GG | CT | CT |
| Chica | GSD | Unaffected | Blood | Frozen | *ab* | GG | GA | AC | GG | TC | CT | GA | TT | CT |
| Nala | GSD | Unaffected | Blood | Frozen | *ab* | GG | GA | AC | GG | TC | CT | GA | TT | CT |
| Boleyn | GSD | Unaffected | Blood | Frozen | *aa* | GG | GG | CC | GG | CC | TT | GG | TT | TT |
| Remus | GSD | Unaffected | Blood | Frozen | *bb* | GG | AA | AA | GG | TT | CC | AA | TT | CC |

**Supplemental Table 3. *ASPA* haplotypes identified in sequenced dogs.**

**A. Six *ASPA* haplotypes were identified.**

| **Haplotype** | **SNP1** | **SNP2** | **SNP3** | **SNP4** | **SNP5** | **SNP6** | **SNP7** | **SNP8** | **SNP9** |
| --- | --- | --- | --- | --- | --- | --- | --- | --- | --- |
| **a** | G | G | C | G | C | T | G | T | T |
| **b** | G | A | A | G | T | C | A | T | C |
| **c** | A | A | C | A | C | T | G | T | C |
| **d** | G | G | C | G | C | T | G | T | C |
| **e** | G | G | C | G | C | T | G | C | T |
| **f** | A | A | A | G | C | T | G | T | C |

**B. The number of each *ASPA* haplotype identified in individual dogs and in six sequenced German Shepherd dogs unrelated to affected pups.**

| **Haplotype** | **Sire** | **Dam** | **Affected Pup AS-1** | **Affected Pup BS-7** | **6 non-affected German Shepherds** | **1 dog of unknown breed** |
| --- | --- | --- | --- | --- | --- | --- |
| **a** | 1 | 1 | 0 | 0 | 5 | 0 |
| **b** | 1 | 0 | 1 | 1 | 4 | 1 |
| **c** | 0 | 1 | 1 | 1 | 0 | 0 |
| **d** | 0 | 0 | 0 | 0 | 2 | 0 |
| **e** | 0 | 0 | 0 | 0 | 1 | 0 |
| **f** | 0 | 0 | 0 | 0 | 0 | 1 |
